# Smaller Gene Networks Permit Longer Persistence in Fast-Changing Environments

**DOI:** 10.1371/journal.pone.0014747

**Published:** 2011-04-25

**Authors:** Jacob W. Malcom

**Affiliations:** Integrative Biology, University of Texas at Austin, Austin, Texas, United States of America; University of Alabama, United States of America

## Abstract

The environments in which organisms live and reproduce are rarely static, and as the environment changes, populations must evolve so that phenotypes match the challenges presented. The quantitative traits that map to environmental variables are underlain by hundreds or thousands of interacting genes whose allele frequencies and epistatic relationships must change appropriately for adaptation to occur. Extending an earlier model in which individuals possess an ecologically-critical trait encoded by gene networks of 16 to 256 genes and random or scale-free topology, I test the hypothesis that smaller, scale-free networks permit longer persistence times in a constantly-changing environment. Genetic architecture interacting with the rate of environmental change accounts for 78% of the variance in trait heritability and 66% of the variance in population persistence times. When the rate of environmental change is high, the relationship between network size and heritability is apparent, with smaller and scale-free networks conferring a distinct advantage for persistence time. However, when the rate of environmental change is very slow, the relationship between network size and heritability disappears and populations persist the duration of the simulations, without regard to genetic architecture. These results provide a link between genes and population dynamics that may be tested as the -omics and bioinformatics fields mature, and as we are able to determine the genetic basis of ecologically-relevant quantitative traits.

## Introduction

Biologists are interested in the diversity of life and the mechanisms permitting maintenance of the diversity. Both evolutionary processes and ecological interactions provide important mechanisms to that end. We would like to more completely unify ecology and evolution into an integrated body that permits scaling from genes up to ecological dynamics, and from ecological dynamics back down to genes; that is, we want to span at least three levels of organization, the genotype, the phenotype, and the environment to elucidate the genotype-environment map. Evolutionary biologists tend to focus on changes in lineages and relative fitnesses, whereas ecologists tend to focus on population changes and absolute fitness. The two fields are joined by the fact that environments are constantly changing and traits must evolve in order to permit population persistence. Van Valen [1] described this as the Red Queen Hypothesis: a population must be constantly running to stay in the same place. Similarly, Anotonovics asserted that ecological change is almost always associated with changes in allele frequencies, i.e., evolution ([2]; tenet 5).

Ecologists increasingly consider that evolutionary change may be an important component of ecological dynamics [3]–[5], which has implications for both basic and applied research. For example, trait evolution can lessen the per-capita impact of predators [6], [7], and ultimately alter community structure [8], [9]. Given contemporary concerns about the impacts of global change [10], we might expect species will need to adapt to novel conditions such as higher temperatures, longer droughts, or novel communities arising from these changes, or else face extinction [11].

The rate at which a trait can evolve is described by a trait's heritability: higher heritabilities confer faster change than lower heritabilities. Heritability is defined as the ratio of genetic (total or additive) variance to phenotypic variance, thus, the greater the genetic variance, the higher the heritability. One of the great advances of the Modern Synthesis, specifically the work of Fisher [12], was the realization that the details of the genetic architecture of a quantitative trait do not need to be known in order to make predictions about a trait's response to selection. However, Crow and Kimura [13] and Bürger [14] showed analytically that the rate of change in genetic variance should be inversely proportional to the number of loci underlying a trait. If the mapping from genotype space to phenotype space is not 1∶1, then there may be a disparity between the rates of change of genetic and phenotypic variance. That is, given the rate of change – number of loci relationship, at a given point in time, we would expect the variance of a small network to have changed more than the variance of a large network, and heritability is affected depending on the rate of change of phenotypic variance. As a result, trait heritability could by systematically affected by the number of genes underlying a trait.

With technological advances in fields such as genomics (and other -omics sciences) and methodological advances in bioinformatics, we can begin to discover the genetic details of phenotypes, including the number of genes underlying a particular trait [15], [16]. One of the results of these novel approaches is the conceptualization of the genotype-phenotype map as a complex network of interacting genes, proteins, and other small molecules [17]–[20]. (Note that other factors, such as other environmental inputs, play a distinct role in the proximate causation of a phenotype [21]. However, because examining the evolution of the trait is the goal of this paper, I focus on the heritable portion of the variation, the genes.) Such a map introduces extensive epistasis as a result of the hierarchy of relationships among genes, and with it, a genotype-phenotype map that is not purely additive. This epistasis may be directional, rather than zero-sum as is assumed in classical analytical models, which means that epistatic variance may be converted to additive genetic variance [22]–[25]. The conversion from epistatic to additive variance in effect hides and reveals standing additive genetic variance, and should alter heritability. Given this departure from the classical additive models, we would like to have a set of expectations for what we should uncover as genomics moves forward. For example, should we expect a priori for some traits to be underlain by fewer genes than others? Do we expect different network topologies for some traits than for others?

The implication of a possible link from gene network characteristics to heritability raises the possibility of systematically linking the genetic architecture of quantitative traits to evolutionary ecological dynamics. Gomulkiewicz and Holt [26] showed analytically that higher trait heritability translates to faster population recovery after a sudden environmental change. Bell and Gonzalez [27] demonstrated the predicted U-shaped recovery path using yeast whose growth media was suddenly changed. A theoretical or computational challenge is to incorporate network representations of the genotype-phenotype map into evolutionary ecology, a problem that has recently begun to be addressed. Three papers stand out as most-similar to the research presented here. Importantly, the authors of each of these papers focused on variation in the density of connections of the underlying network, which, due to the computational complexity, limited the size of the networks they examined. Frank [28] described the evolution of a network underlying a trait that needed to pass through two distinct developmental phases and found that intermediate network connectivity resulted in the greatest robustness. Kimbrell and Holt [29] used a model similar to Frank's and found that colonization of a novel patch from a source patch was maximized when gene network complexity was minimized. Repsilber and colleagues [30] modeled small gene networks (3–10 genes) of varying connectivity and found that smaller networks result in faster evolution.

Malcom took the opposite approach and simplified network connectivity while examining the evolution of a trait underlain by networks of 16–256 genes to test the effects of genetic architecture on trait heritability in a static environment and population recovery after a sudden environmental change [31]. He found that smaller, scale-free networks conferred higher heritability and faster population recovery than larger, random-topology networks. A natural extension of these results is to hypothesize that smaller networks permit longer persistence times in constantly-changing environments.

In this contribution I use simplified gene network connectivity, but assume that environmental change is constant and fluctuates between a maximum and minimum through time (directional selection). I test two basic hypotheses: first, the genetic architecture (i.e., number of, and functional relationship among, genes) of a quantitative trait plays a large role in determining the trait's heritability under constant directional selection. Second, the genetic architecture, by way of heritability, affects the persistence times of populations in a fluctuating environment. I find that both hypotheses are supported, with a caveat that the rate of environmental change is very important. There is a strong interaction between network size and the rate of environmental change such that small-network populations persist longer when the environment changes rapidly, but populations in slowly-changing environments persist indefinitely without respect to network size. I discuss how these results refine those of Malcom [31], and how, in conjunction with prior research focused on network connectivity, they provide a set of expectations requiring empirical testing.

## Results

**Figure 1:**
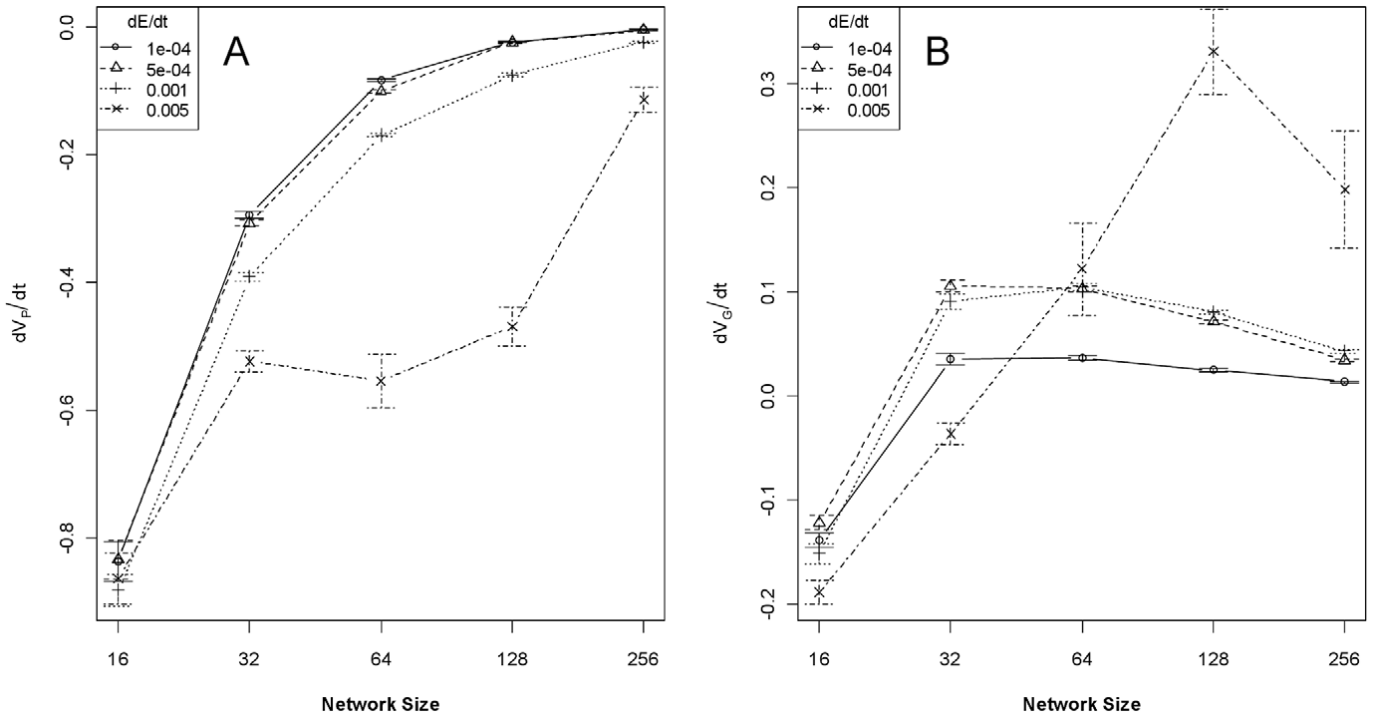
Rates of change of phenotypic and genetic variance as a function of network size and rate of environmental change. Panel A shows the rates of change (±95% CI) of phenotypic variance during the first 250-generations of simulations, conditional on different-sized gene networks and different rates of environmental change. Panel B shows the rates of change of genotypic variance (±95% CI) during the same time period. Selection at the phenotype-environment interface has a disproportionate affect on V_P_ compared to V_G_ in all but the smallest networks.

Differences in genetic architecture result in differences between the rates of change of phenotypic and genotypic variance (dV_P_/dt and dV_A_/dt, respectively; Table 1). dV_P_/dt in a fluctuating environment depended primarily on the size network underlying the trait and the rate of environmental change (Figure 1A). The AIC-best model (i.e., the model with the lowest Akaike's Information Criterion value) included all terms and first-order interactions, making interpretation very convoluted. A much reduced model (ΔAIC >300) used only network size and rate of environmental change still explained 68% of variance in dV_P_/dt. Pairwise contrasts (Tukey HSD) showed that all network size contrasts, with the exception of 64- and 128-gene networks, were significantly different in their effect on dV_P_/dt. In contrast, dV_A_/dt varied much less according to the specifics of network architecture, with the exception at the smallest network size (Figure 1B). Interestingly, V_A_ tends to actually increase for networks with >32 genes as epistatic variance is converted to V_A_. The most readily-interpretable model (ΔAIC  = 11) for dV_A_/dt used only network size and rate of environmental change as predictors, but explained only 25% of the variance. Tukey HSD comparisons showed that dV_A_/dt for 16-gene networks was significantly different from all other network sizes, but there were no other significant differences. The ‘atypical’ mean estimates and large confidence intervals for 64–256 gene networks at the highest rate of environmental change reflects the rapid extinction of populations with these combinations of network size and rate of change.

**Table 1:** Factors affecting the rates of change of genetic and phenotypic variance during the first 250-generations in a constantly-fluctuating environment.

Response	Predictor	% Var.	*P*-value
Variable		Explained	
dV_P_/dt	Network Size	57	<2.2e^−16^
	dE/dt	8	<2.2e^−16^
	*n* * dE/dt	4	2.29e^−7^
dV_G_/dt	Network Size	16	<2.2e^−16^
	dE/dt	2	0.013
	*n* * dE/dt	7	0.0001

*dV_P_/dt* is the rate of change of phenotypic variance; *dV_G_/dt* is the rate of change of genetic variance. *n* refers to network size; *dE/dt* is the rate of environmental change.

**Figure 2:**
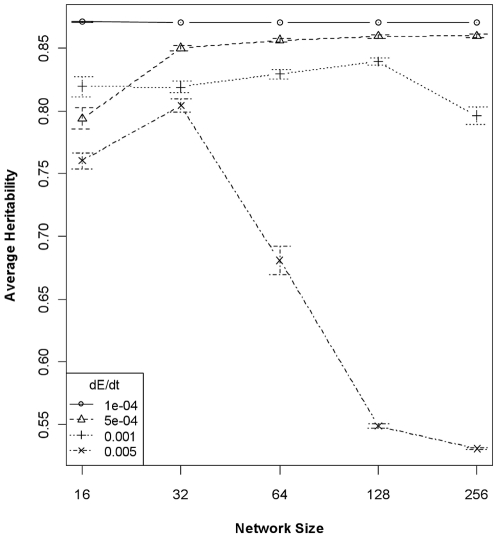
Average heritability (±95% CI) of the quantitative trait as a function of network size and rate of environmental change. The differential impacts of selection on rates of change of phenotypic and genotypic variance results in higher heritabilities for trait underlain by small (and scale-free) networks. As importantly, the rate of environmental change alters heritabilities, with higher rates of change resulting in systematically lower heritability.

The differences in rates of change of variance components translated to systematic alterations of heritability, at least at some rates of environmental change. The global model relating all predictors to average trait heritability over the course of each simulation run possessed the lowest AIC score and R^2^ = 0.93. The reduced model that used only network size and rate of environmental change as predictors had a much higher AIC score (ΔAIC ∼ 300), but still explained 78% of the variance in average heritability (*P*<2.2e^−16^; Table 2). Network size alone accounts for little variance, but when considered with the rate of environmental change, a clear interaction emerges: at slow rates of environmental change all network sizes converge on high heritability, but heritability declines with increasing network size at the fastest rate of change (Figure 2). That is, proportionally more additive variance is removed from large-network populations relative to the amount of phenotypic variance at high rates of environmental change. Tukey HSD contrasts showed the effects on heritability of most network contrasts, and all rates of environmental change, to be significantly statistically different at α = 0.05. Many interactions between network size and rate of environmental change were not significantly different, as is evident in Figure 2. The preceding results are useful, but do not reveal the dynamics of the evolution of variance components and heritability through time. All variance components are very similar at the end of the 2,000-generation simulation, but significantly lower variance values for larger networks in the early stages of the simulations has a large impact on whether or not the population will survive long enough for high heritability to evolve (Figure S1).

**Table 2:** Primary factors influencing trait heritability averaged over the existence of populations.

Predictor	% Var.	P-value
	Explained	
Network Size	5	0.001
dE/dt	52	<2.2e^−16^
*n* * dEdt	21	1.75e^−12^

*n* refers to network size; *dE/dt* is the rate of environmental change.

**Figure 3:**
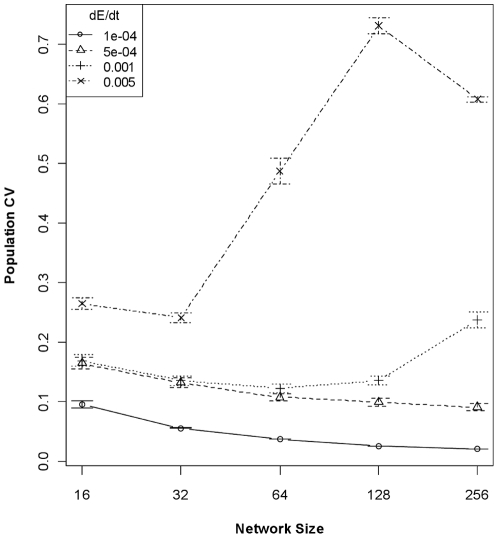
Population size Coefficient of Variation (CV; ±95% CI) as a function of network size and rate of environmental change. The amount of variation in a population time-series is positively related to network size when the rate of environmental change is fast, but negatively related when the rate of change is slow. The higher stochasticity of a population, the greater the likelihood of extinction.

**Figure 4:**
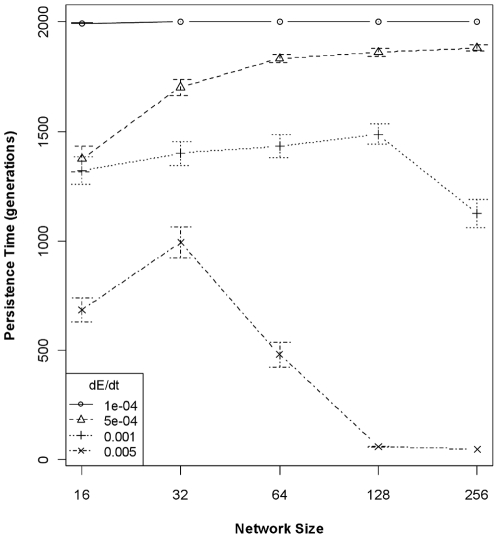
Population persistence times (±95% CI) as a function of network size and rate of environmental change. The generally negative relationship between network size and persistence times is evident at the highest rate of environmental change (0.005 units/generation). At the slowest rate of environmental change (1e^−4^ units/generation), however, the relationship with network size is absent: the environment is changing slowly enough that all networks can adapt sufficiently fast.

The joint effects of genetic architecture and rate of environmental change on trait heritability translate directly to differences in levels of population variation through time and population persistence times in a fluctuating environment. At high rates of environmental change, population size coefficient of variation (CV) increased with increasing network size; there was little relationship between network size and CV at intermediate rates of environmental change; and CV was slightly negatively related to network size at the slowest rate of environmental change (Figure 3). These relationships are reflected in population persistence times. The global model relating population persistence to interactions among all predictor variables possessed the lowest AIC by nearly 30 points, but the residuals were strongly kurtosed. The reduced model employing only network size and rate of environmental change possessed an AIC score >100 points higher than the global model, but the model residuals were normally distributed and the reduced model still explained 66% of the variance (*P*<2.2e^−16^; Table 3). Network sizes appear to be “matched” to a given rate of environmental change, such that when the environment is changing rapidly, smaller networks confer an adaptive advantage that translates to longer population persistence (Figure 4). However, when the rate of environmental change is very slow, populations with any size network encoding the critical trait are able to adapt and populations tended to persist the duration of the simulation.

**Table 3:** Primary factors influencing population persistence times in a fluctuating environment.

Predictor	% Var.	P-value
	Explained	
Network Size	1	0.001
dE/dt	58	<2.2e^−16^
n * dEdt	7	1.75e^−12^

*n* refers to network size; *dE/dt* is the rate of environmental change.

**Figure 5:**
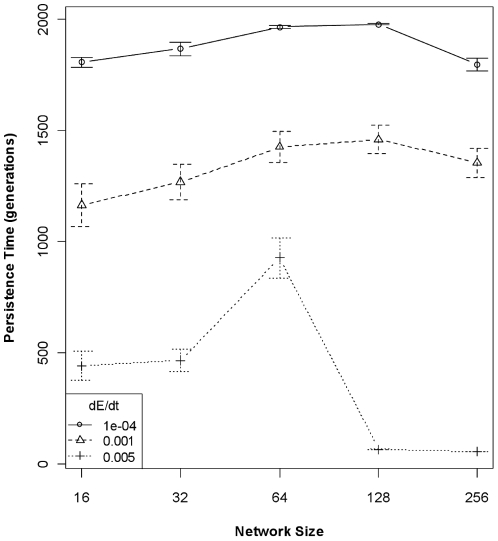
Population persistence times (±95% CI) in fluctuating environments, as a function of network size and rate of environmental change, when controlling for additive variance. Populations exhibit a strong network-by-rate of environmental change (dE/dt) effect: smaller networks tend to perform better than large networks at high rates of environmental change, but population performance is essentially identical when the rate of environmental change is very slow. See the text for a discussion of the high persistence time values for 64-gene networks at dE/dt  = 0.005.

An implication of more genes underlying variation in a particular trait is systematically lower additive genetic variance for larger networks at simulation initiation [31]. By virtue of these differences we would expect differences in persistence time without some canalization so that different-size networks have equivalent levels of variance. To address this aspect, I ran another set of simulations in which the environment was held constant until the population achieved an additive genetic variance of 5, 10, or 15. Environmental fluctuations began at the same rates in the experiments above once the ‘trigger’ level of V_A_ was reached. When the additive variance is approximately the same between network sizes, smaller network populations tend to persist longer in faster changing environments and there is little difference in persistence to the end of the simulation when the rate of environmental change is slowest. However, the strongest determinant of persistence is clearly the rate of environmental change (Figure 5). The higher average persistence time of 64-gene networks is an artifact of simulation truncation: the time-to-start of fluctuations was much lower for 64-gene networks than for 16- and 32-gene networks. It is likely that if these simulations had continued beyond 2000 generations the 16- and 32-gene populations would have persisted longer than the 64-gene populations. Variance partitioning (Table 4) quantifies the importance of the rate of environmental change. It also shows the role of mutation rate, and the mutation-by-network size interaction (i.e., mutational variance), in shaping persistence time.

**Table 4:** Primary factors influencing population persistence times when fluctuations start at a given level of additive variance in the population.

Predictor	% Var.	P-value
	Explained	
Network Size	2.3	5.18e^−12^
Mutation rate	12.1	<2.2e^−16^
dE/dt	60.5	<2.2e^−16^
n * dEdt	3.8	2.48e^−16^
Mutation * dE/dt	9.4	<2.2e^−16^

*n* refers to network size; *dE/dt* is the rate of environmental change.

## Discussion

Biology is approaching the stage at which data can be gathered from the level of entire genomes up through communities. Successful integration across levels of organization will require bridging at least three distinct levels: the genotype, the phenotype, and the ecotype (i.e., environment). The processes of gene duplication and loss [32] provide a mechanism by which the gene networks underlying quantitative traits may evolve (both in size and topological organization), and potentially alter the speed at which the trait can evolve. This, in turn, has the potential to limit the environments in which a species can persist when the environments are constantly changing. We have analytical expectations of a relationship between genetic variation and population dynamics in changing environments [33], [34]. For example, Bürger and Lynch [34] explored the relationships between rates of environmental change, population persistence, and genetic variance analytically and with a 50-locus, additive model. Here I build upon their work—and previous research focused on variation in network connectivity rather than size—to investigate how network size and basic topology influence quantitative trait heritability and population persistence in a changing environment. I find that network characteristics and the rate of environmental change interact to shape trait heritability, which ultimately alters population persistence times.

All network sizes have time to evolve to a high average heritability under the relatively weak selection imposed by a slowly changing environment. This is the conclusion from classical quantitative genetics [35]: there is no relationship between the number of underlying loci and heritability. This results from the fact that there is no variance at non-terminal genes in the network (i.e., upstream, controlling genes) and the GPM is essentially purely linear. At high rates of environmental change, however, populations in which the trait is underlain by a large network are much slower to adapt, go to extinction more quickly, and are not able to evolve high heritability. As such, a relationship between network size and heritability is maintained, as in simulations where the environment is modeled as a static value with a single, sudden change [31]. The result is that the rate of environmental change interacts with the species' genetic architecture for the limiting trait to constrain or facilitate the evolution of the trait's heritability. This result is consistent with, although examined in a rather different context than, the findings of Price and Schluter [36]. They showed that high environmental variance should depress heritability even when substantial additive genetic variation is present.

The effects of genetic architecture and environmental change cascade to systematically alter population persistence times. Only populations where the trait is underlain by small networks persist for even several hundred generations when the rates of environmental change are high. In contrast, when the rate of environmental change is very slow, populations persisted the duration of the simulations regardless of the underlying genetic architecture. Genetic variance of internal genes is lost over these longer time periods resulting in the purely linear GPM. Given that smaller networks result in greater genetic variance because the variance contribution of each gene is greater, this is essentially the same result found by Lande and Shannon [37] using analytical models of additive genotypes.

Building from the literature on the diversity-stability hypothesis [38], Agashe used *Tribolium* to show that increased heritable variation in a population resulted in increased population dynamic stability [39]. Here I have found that population stability (CV) tends to be related to higher additive genetic variance, but that it is conditional on the background rate of environmental change. Willi and Hoffmann [40] found *Drosophila* population persistence correlated with genetic variability (and demographic parameters), with greater variability resulting in longer persistence. The network-to-persistence time hypothesis could be tested by combining GPM estimates with population experiments such as those of Agashe or Willi and Hoffmann.

While range expansion was not modeled here, prior research has indicated that the heritability of a limiting trait can play a strong role in determining range limits [41]. Patterson and Stone noted nearly 70 years ago that the range of *D. melanogaster* in North America had expanded north faster than the range of *D. simulans* [42]. This information taken in conjunction with the estimates of cold resistance heritability in several *Drosophila* species by Kellermann and colleagues [43] suggests a causal chain of higher heritability resulting in greater population persistence, which then results in faster range expansion. There may, in fact, be a feed-forward process in that the continued range expansion will permit the maintenance of even greater genetic diversity in a patchy landscape, and contribute to increased heritability. Network size (and topology) evolution could contribute to the process. The link between size and topological characteristics of networks underlying range-limiting traits in various species could be tested in a straightforward, if correlational, manner.

The differences in population persistence given different network characteristics at different rates of environmental change suggests a novel axis of species sorting. Species sorting is a specific model of community assembly that proposes species sort according to their ‘preferred’ habitats [44]–[46]. Here, rather than suggesting species possess fixed traits which are environmentally filtered, it appears that species could assort according to the rate of environmental change. These differences in rates of change could either be spatial or temporal. We can hypothesize that such population-level effects of genetic architecture could be extended further. For example, Urban and colleagues investigated the role of heritability on community assembly dynamics and found that different heritabilities affect the ability of species to colonize and monopolize patches in a metacommunity [9]. By extension, gene networks that contribute to variation in heritability may provide a mechanistic basis of scaling from genes to communities.

Given the importance of the rate of environmental change in interacting with genetic architecture to potentially shape heritability and population persistence times, what should the reader make of the rates that have been examined here? The values were not taken from the literature for any long-period cyclical environmental variable (e.g., ENSO). Instead, the goal was to explore a parameter space of rates of environmental change to see if an interaction with network characteristics was apparent. Such an interaction was recovered. The inference should therefore be that the genetic architecture is important in the context of the rate of environmental change relative to some faster or slower rate of change, but we should not expect these values to be empirical estimates. The simplicity of a Boolean network further precludes a direct application to reality. We should only state from these results that relative network sizes, say, compared between species, could be an important component of explaining population persistence in fluctuating environments.

As with any model, the system investigated here is a simplification of reality. The network structure and dynamics are simplified for computational tractability in the present model, and future computational research should consider combining the larger network sizes (such as those here) with the more complex topology as considered by others [28], [29]. Here, I have considered only a single trait and ignored pleiotropic effects, which are well-known to influence rates of trait evolution [47]; future work should investigate the intersection of networks and pleiotropy. Lastly, only a single-species is considered here, but real species exist in communities where heterospecifics also evolve as they compete with, prey upon, facilitate, or parasitize a focal species [48]. However, even with these simplifications, the model is useful because it suggests new relationships between gene networks and ecological dynamics. Furthermore, the conclusions establish basic hypotheses to be tested empirically.

The basic conclusion of this paper is that the genetic architecture of a quantitative trait—that is, the size and topology of the underlying network—interacts with the rate of environmental change to alter trait heritability, which in turn effects persistence times. In particular, populations whose limiting trait is underlain by smaller, scale-free networks persist longer in fast-changing environments, and quickly gain higher trait heritability as a result. In contrast, populations persist indefinitely regardless of the details of genetic architecture when the rate of environmental change is slow, and even large-network species can achieve high trait heritability. Either rejecting or supporting these conclusions empirically will lead to a better understanding of the evolution and ecology of species and communities.

## Methods

### Model Presentation

I focus on individuals of a single species living in a single patch with an environmental variable against which each individual's trait is tested. This variable is a “driver”, i.e., a variable whose value is not affected by the presence or activity of individuals in a patch. Examples of drivers include temperature and pH. In these simulations the environment can take a value between 0 and 140, and was initialized at 70 in all simulations. The environment begins changing at a constant rate—either 5e-3, 1e-3, 5e-4, or 1e-4 units per generation—immediately upon simulation initiation.

Individuals possess a single quantitative trait that maps to the environmental variable. For the three environmental driver examples above, this might include thermoregulatory ability, or the ability to regulate osmotic balance or pH. The trait is encoded by a directed Boolean network of 16, 32, 64, 128, or 256 genes, the state of each determined dynamically (see below). The topology of the network is initiated as either random (no preferential attachment) or scale-free (with preferential attachment) in its out-degree distribution [19]. Randomly-connected networks show an approximately Poisson degree distribution, whereas scale-free networks exhibit an power law degree distribution [19]. I use a lottery model algorithm, i.e., the probability of an existing gene acquiring a connection to a new gene is proportional to the number of existing connections, to form the scale-free networks [49].

**Figure 6:**
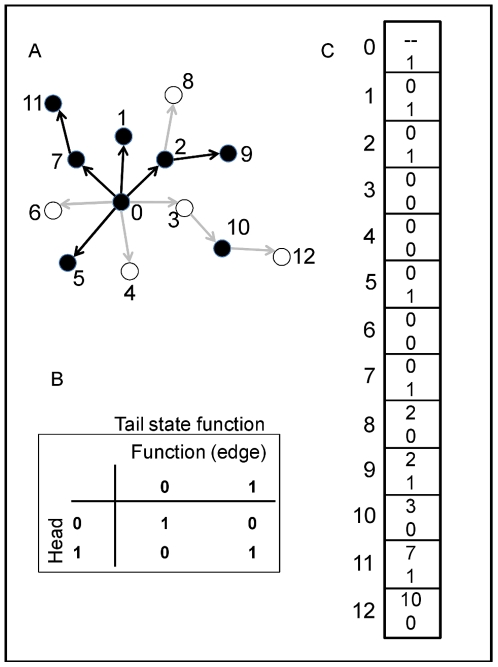
An example network, functional map, and chromosome. Panel A shows an example 13-gene Boolean network. Black nodes are up-regulated (“on”; state  = 1) genes and white nodes are down-regulated (“off”; state  = 0). If an edge connecting two nodes is black, the “head” gene (upstream) activates the “tail” gene (downstream), and if an edge is gray, the head represses the tail gene. Panel B provides the functional map; for example, if the head gene is “off” and the edge connecting the head and tail genes is an activator, then the tail gene is off (upper-right quadrant). Panel C shows the chromosome corresponding to the network in Panel A. Each block represents a gene (numbers along the left-hand side); within each block, the top number defines the “head” (i.e., immediately-upstream) gene while the bottom number defines the functional relationship (e.g., if 0, then the head gene is a repressor).

At the start of a run, every individual's network is randomly determined (as guided by the constraints of topological specification); with these relatively small populations, it is very unlikely that any two individuals possess the same exact network at simulation initiation. The binary state [0, 1] of each gene in the network except the upstream-most is determined by comparing the state of the gene immediately upstream to the functional relationship of the gene pair (Figure 6a, encoded by chromosome of 6c). The state of the upstream-most gene is determined randomly for each individual at simulation initiation, and is then inherited for subsequent generations. Some genes may act as repressors and others as activators, and the state of the downstream gene is determined by the match or mismatch between the state of the upstream gene and the function (Figure 6b). For example, if the upstream gene is “on” (state  = 1) and is a repressor (function  = 0), then the downstream gene takes the “off” state (state  = 0). Alternatively, if the upstream gene state is 0 and it is a repressor, then the downstream gene takes the “on” state. Each gene except the basal-most has a single input to ease computational requirements (the number of calculations increases according to 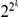 with *k* inputs [28]), but may have one or more outputs (i.e., may be pleiotropic). All network information is stored on a single chromosome consisting of two parts (Figure 6c). First, the topology is defined by a “tails list” of the downstream genes; the “heads list” (the controlling, upstream genes) is inferred from the index position of each tail list element. The relationship between heads and tails genes is randomly determined at the start of a simulation run, but, as noted above, the out-degree distribution is constrained by the scale-free versus random topological assignment. Figure 6a is an example 13-gene network whose states have been calculated given the information from the chromosome in Figure 5c.

Each individual's phenotype is determined by summing the states of all terminal genes in the network, i.e., genes with out-degree  = 0, and scaling the value to the range of the environment ( = 140). So, for example, the network in Figure 6a possesses eight terminal genes, four of which are “on”, thus the individual possesses a phenotype of 70 ( =  (140/8) * 4). I am thereby assuming that there are no biochemical limits given a particular network size; individuals with a 16-gene network can approximate a phenotype of 140, as can individuals with a 256-gene network. The consequence for this re-scaling is that smaller networks have lower resolution than larger networks, which is a reasonable assumption given that dividing any particular task among fewer actors will result in lower overall accuracy. I stored the phenotypes of each individual's parents and used mid-parent regression to estimate the trait's heritability in the population. Additive genetic variance was derived by multiplying the phenotypic variance by the heritability.

Each individual's phenotype is translated to a fitness relative to the environment using a Gaussian function of the form,
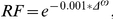
where Δ is the absolute value of the difference between the environment and the phenotype, and ω is a value that changes the breadth of the selection function. I varied ω from 1.5 (high tolerance for a phenotype-environment mismatch) to 2.5 (low tolerance for a phenotype-environment mismatch) in the simulations. In this way I assume that the environmental effect is absolute and the phenotypic variance of the population plays no role in how an individual is selected. Each individual's *RF* does not affect the number of offspring produced, but does affect the probability that an individual will survive to reproduce.

Individuals are sexually-reproducing hermaphrodites who mate at random. The number of offspring from a mating is determined by drawing a random value from a Poisson distribution with λ = 1.5. Gametes undergo recombination during a diploid meiotic stage to create an offspring chromosome that is a mixture of parental alleles, which in this model are the tails list and the functional relationships. The first element of the offspring chromosome is chosen from the first element of one parent, then subsequent elements are taken from the same parent until a random uniform number less than the recombination rate (*r* = 0.05 or 0.5) is drawn, at which point the element is drawn from the opposite parent. This continues the length of the chromosome. Mutation, as determined by testing a uniform random number against the mutation rate (1e^−4^ and 1e^−6^) for each chromosomal element, occurs after the new chromosome is created. Although these mutation rates appear high, as noted by Frank [28], because the trait is directly related to fitness, the effective mutation rate is about one order of magnitude lower. All mutations are non-synonymous and may affect either the controlling function of a gene (an activator mutates to suppressor) or the relationship to another gene (i.e., alter network topology).

Death occurs after reproduction in three stages. First, all parents are killed to prevent over-lapping generations. Next, the new generation is culled according to each individual's relative fitness: if the *RF* is less than a uniform random number, then the individual dies. Last, a carrying-capacity is enforced by randomly killing individuals to bring the population below K  = 500.

### Analysis

The experiments were a full factorial design using five network sizes, two network topologies, two recombination rates (0.05 and 0.5), two mutation rates (1e^−4^ and 1e^−6^), and the four rates of environmental change, replicated three times for a total of 480 simulations. (I also ran 480 simulations of an earlier version of the model, in which the recombination code was insufficient, and the results of that run were nearly identical to those presented here.) The simulations continued for 2000 generations or until the population went extinct, whichever occurred sooner. I considered four response variables: the rate of change of phenotypic variance, the rate of change of genotypic variance, average trait heritability over the duration of each simulation, and population persistence. I extracted the rates of phenotypic and genetic variance change during the first 250 generations of each of the 480 simulations using a liner regression of time on genetic and phenotypic variance. I then used linear regressions to assess the influence of each predictor (characteristics of genetic architecture plus the rates of environmental change) on each of the response variables. In all analyses, predictor variables were factors, rather than continuous variables, thus obviating a need for nonlinear model terms. I used Tukey's HSD to calculate corrected pairwise tests [50]. For some analyses, a full-interaction model resulted in far too many terms to be readily interpretable. I therefore used Akaike's Information Criterion (AIC) to determine how different the best interpretable model was from the AIC-best model [51]. All statistical analyses were completed in R 2.10 [52].

## Supporting Information

Figure S1 An example of change in variance components and heritability over 2,000 generations. The mean additive genetic variance, phenotypic variance, and heritability of the ecologically-important trait regulating the simulated species' population dynamics, when the rate of environmental change is slow (dE/dt  = 0.0001 units per generation). V_A_ is derived from the directly-measured parameters heritability (from mid-parent regression) and phenotypic variance. Even though variance components for each network size converge by 2,000 generations, larger networks start with lower variance and are not able to adapt fast enough to survive long enough to evolve the beneficial, higher heritabilities when dE/dt is high.
(0.65 MB TIF)
