# Supplementary figures and images for: Smaller Gene Networks Permit Longer Persistence in Fast-Changing Environments
Source: PLoS One. 2011 Apr 25;6(4):e14747. doi: 10.1371/journal.pone.0014747 (PMC3081814; doi:10.1371/journal.pone.0014747)

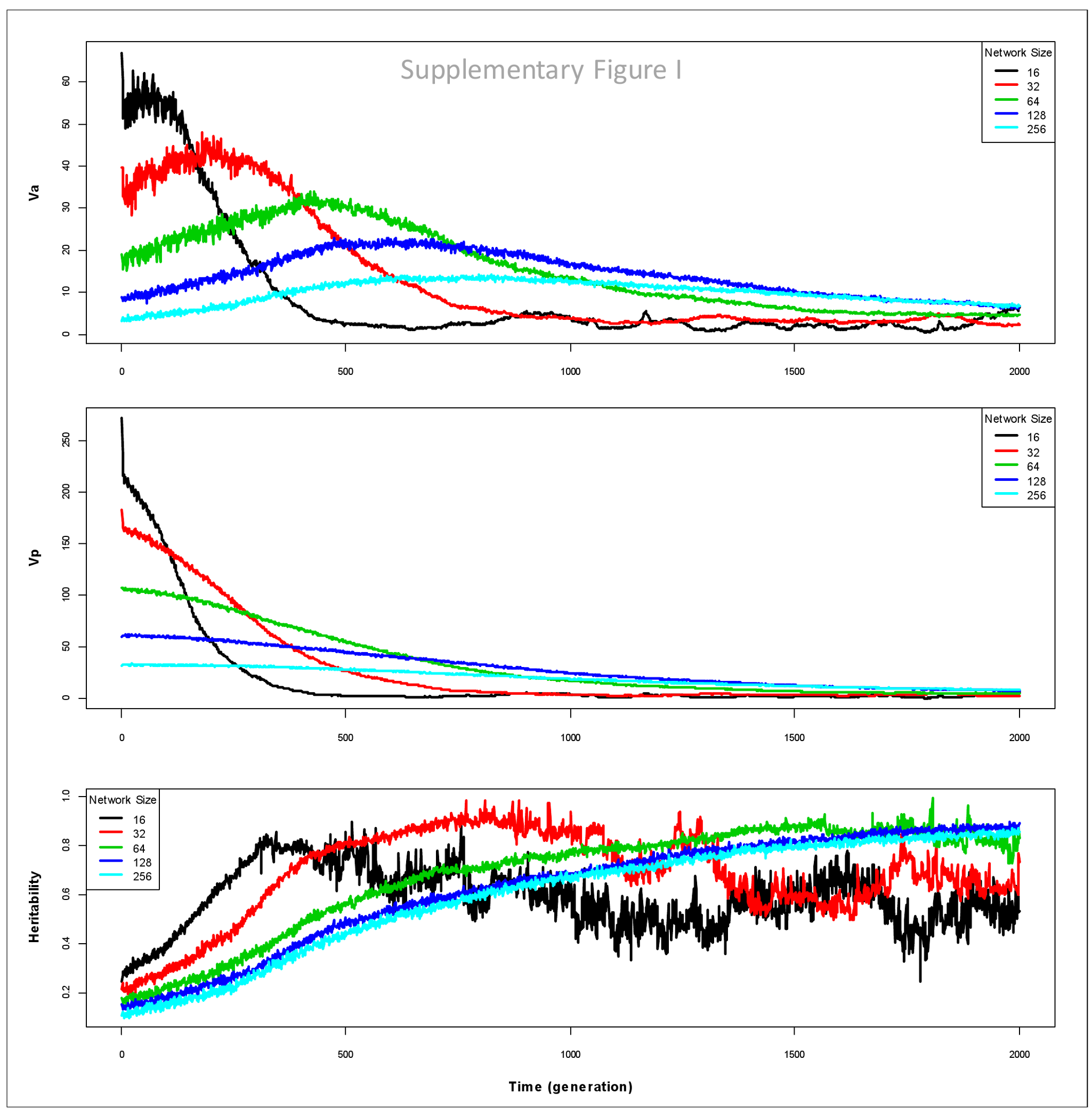

Supplement: Figure S1 — An example of change in variance components and heritability over 2,000 generations. The mean additive genetic variance, phenotypic variance, and heritability of the ecologically-important trait regulating the simulated species' population dynamics, when the rate of environmental change is slow (dE/dt = 0.0001 units per generation). VA is derived from the directly-measured parameters heritability (from mid-parent regression) and phenotypic variance. Even though variance components for each network size converge by 2,000 generations, larger networks start with lower variance and are not able to adapt fast enough to survive long enough to evolve the beneficial, higher heritabilities when dE/dt is high. (0.65 MB TIF) [file pone.0014747.s001.tif]
